# Hierarchical State-Space Estimation of Leatherback Turtle Navigation Ability

**DOI:** 10.1371/journal.pone.0014245

**Published:** 2010-12-28

**Authors:** Joanna Mills Flemming, Ian D. Jonsen, Ransom A. Myers, Christopher A. Field

**Affiliations:** 1 Department of Mathematics & Statistics, Dalhousie University, Halifax, Canada; 2 Department of Biology, Dalhousie University, Halifax, Canada; University of California, United States of America

## Abstract

Remotely sensed tracking technology has revealed remarkable migration patterns that were previously unknown; however, models to optimally use such data have developed more slowly. Here, we present a hierarchical Bayes state-space framework that allows us to combine tracking data from a collection of animals and make inferences at both individual and broader levels. We formulate models that allow the navigation ability of animals to be estimated and demonstrate how information can be combined over many animals to allow improved estimation. We also show how formal hypothesis testing regarding navigation ability can easily be accomplished in this framework. Using Argos satellite tracking data from 14 leatherback turtles, 7 males and 7 females, during their southward migration from Nova Scotia, Canada, we find that the circle of confusion (the radius around an animal's location within which it is unable to determine its location precisely) is approximately 96 km. This estimate suggests that the turtles' navigation does not need to be highly accurate, especially if they are able to use more reliable cues as they near their destination. Moreover, for the 14 turtles examined, there is little evidence to suggest that male and female navigation abilities differ. Because of the minimal assumptions made about the movement process, our approach can be used to estimate and compare navigation ability for many migratory species that are able to carry electronic tracking devices.

## Introduction

Electronic tracking technologies have enabled the migratory movements for a great variety of animals [1], [2] to be determined. In particular, remotely sensed data from marine animals reveal remarkable migration patterns that were previously unknown [2]–[4]. This leads to questions of how developed an animal's navigation ability must be in order to successfully complete long-distance migration and how individuals may vary in this ability.

To answer such questions using ubiquitous electronic tracking data, we require statistical methods that can deal with typical features of these data such as irregularly-timed and serially correlated observations with non-Gaussian errors that vary through time. A lack of suitably effective and routine estimation methods for these kinds of data has made it difficult for ecologists to fully utilize such tracking data. However, state-space models have recently emerged as a key tool for analyzing tracking data [5], [6]. State-space models allow us to capture an animal's movement behaviour as well as model the two types of stochasticity that are typical of observed movement pathways: (i) stochastic deviations from a deterministic movement model (process uncertainty), and (ii) stochastic deviations from an animal's true location (observation error).

Our work focuses on estimating the navigation ability of organisms as they move through their environment. Flemming *et al.* [7] developed a statistical model with minimal assumptions to describe how well a migrating animal knows its true position. The idea stems from the pioneering work of Kendall [8], who developed analytical and simulation models to describe the ability of Manx Shearwaters (*Puffinus puffinus*, Brunnich 1764) to successfully navigate a route spanning the Atlantic Ocean back to their breeding colony. Kendall suggested that animals have a “circle of confusion” within which they cannot know their position while navigating via indirect cues. In his model the radius of the circle of confusion must be smaller than the distance at which an animal can detect, eg. by sight or olfaction, and orient directly toward its destination; otherwise the animal will not reach its destination efficiently. This implies that migrating animals' navigation abilities need only be good enough to put them within detection range of their ultimate destination.

Whereas Kendall [8] focused on estimating expected times to cross into this detection range, we focus on estimating the size of an animal's circle of confusion as a means of quantifying navigation ability. The larger the circle of confusion, the less developed the navigation ability. Of particular interest is the estimation not only of individual circles of confusion but also of an overall circle of confusion to describe navigation ability at a group or population level. To achieve this, we fit our state-space model to tracking data in a hierarchical Bayes framework. Hierarchical Bayes methods are gaining popularity in the ecological literature [9] as tools for combining information from multiple sources (meta-analysis) and for inference and prediction of complex relationships. In addition this framework allows us to formally test hypotheses of interest using the Minimum Posterior Predictive Loss Statistic [10].

We illustrate how the circle of confusion can be estimated at both individual and population levels. A hierarchical Bayes state-space model is fit to Argos satellite tracking data on 14 leatherback turtles (*Dermochelys coriacea*, Linn. 1766) during their southward migration from Nova Scotia, Canada [2]. Specifically, we test hypotheses about the difference in navigation ability between males and females and whether turtles compensate for large course deviations by “re-calculating” the most direct route to their destination. The leatherback is a cosmopolitan marine species that undertakes the most extensive migrations of all sea turtles [11]. This species is now critically endangered [12] and there is great interest in understanding its migratory behaviour and navigation ability [2], [13].

## Methods

### The Circle of Confusion model

**Figure 1:**
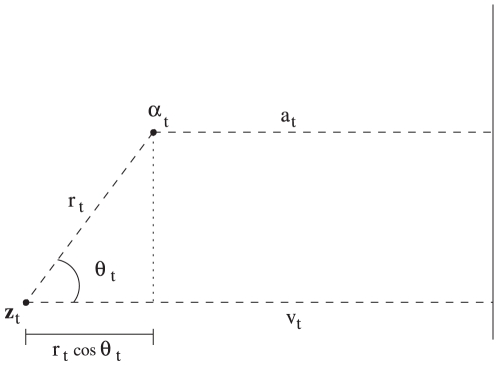
Geometry of relationship between observed deviation 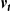 and unobserved deviation 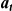. Note that the corresponding distance along the GC route is assumed to be linear as it is so small.

Our interest is in an animal's ability to follow a particular migratory route. We consider positions of an animal at equally spaced points in time, 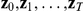 as it moves from an initial position, 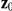, to a final destination point, 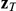. These positions are derived from satellite tags and are subject to error. As a result, we assume that this observed time series of location coordinates derives from an unobservable state process 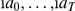 describing the *true* pathway of the animal. Here we presume that the animal tries to follow the most direct route to its destination, i.e. a Great Circle (GC) route while noting that other presumed routes could easily be used. As in Flemming *et al.* [7], we view the true locations 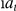 as representing the animal's *best guess* at where it is relative to this desired pathway. Note that it is straightforward to superimpose the GC route between any starting point 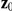 and ending point 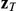 as it is simply the shortest distance on the earth's sphere between these two points. We define 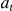 as the distance from 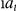 to the nearest point on the GC route. Similarly, 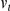 is defined as the distance from 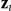 to the nearest point on the GC route. Both 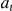 and 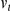 can be regarded as deviations from the GC route, only the latter of which are observable. Figure 1 illustrates the relationship between these deviations. Note that the difference between the deviations 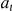 and 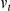 describes the measurement error of the deviations and can be expressed as 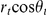.

If the animal is to one side of the GC route at one particular point in time it is likely that it will also be on this side of the GC route at the next point in time. This behavior suggests that the 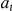 are not independently and identically distributed but rather possess some dependence structure. Here we assume that they can be represented by a first order autoregressive process with correlation 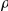 and errors 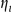. In summary, we have arrived at a state-space model for the observed deviations 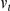 consisting of the following two equations:
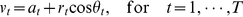
(1)

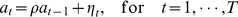
(2)where the measurement errors 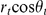 are independently and identically distributed with mean 0 and unknown variance 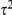, and the process errors 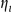 are independently and identically distributed with mean 0 and unknown variance 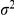. In contrast to the frequentist approach taken in Flemming *et al.* [7], we consider here a fully Bayesian framework for the state-space model described above. Such a framework naturally accommodates prior information and allows us to formulate a hierarchical model for a collection of animals in a straightforward manner.

The overall variance of the autoregressive model encapsulated in the state-space model (Equations (1) and (2) above) is 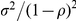. As discussed in detail in Flemming *et al.* [7] this quantity is not only interpretable from a statistical perspective but also from a biological one, leading us to define the circle of confusion as 
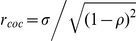
, where 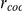 is interpreted as a circle with radius 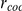 drawn about the animal's location within which it is unable to determine its location precisely. Note that 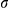 is indicative of the random variation of any animal around a localized path which is determined to a degree by the parameter 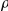, itself indicative of autocorrelated movement. High variability around localized paths reflects poor navigational skills (and consequently a large 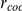), if it is assumed that the animal is trying to efficiently navigate a path. Hight levels of autocorrelation in distance from a GC route also suggest poor navigational skills because the animal is not correcting its path towards a GC route when it bounces off the track. The smaller the 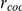, the better the navigation ability of the animal. This measure of precision has a quantitative analogue in confidence interval: 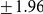 times this quantity is the animal's 95% CI of where it is located.

### Hierarchical Bayesian Framework

Our goal is to infer among-individual variation in navigation ability. Such variation implies that there is an underlying, biologically meaningful distribution of navigation abilities. The framework discussed in Flemming *et al.* [7] only allows a state-space model to be fit to an individual pathway. However by formulating this state-space model within a hierarchical Bayes framework we are able to analyze data from a collection of individuals thereby formally incorporating the idea of among-individual variation.

It is straightforward to formulate a hierarchical model by placing hyper-priors on the priors of the model parameters. In so doing the corresponding parameters become random variables. By letting 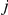 index the individual, 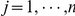, we arrive at a collection of 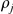
's (and 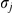
's), one for each individual. A beta distribution with parameters 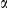 and 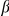 is then transformed to obtain a prior distribution for each 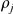 that is necessarily bounded (between -1 and 1). With uniform priors for both 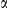 and 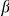, our formulation for the 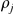 is complete and one is able to estimate the collection of 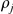
's as well as the mean and variance of their distribution. Following the suggestion of [14], we use a half-normal prior distribution (with mean 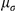 and variance 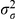) for the process error standard deviations, 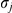,and uniform distributions for the hyper-priors. A uniform prior distribution is also used for 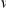, the degrees of freedom of the process error distribution (as discussed later in the data section). By specifying an informative prior distribution for 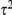 we can easily incorporate measurement error information. Note that a hierarchical structure for 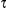 is not appropriate as the measurement error distribution is assumed to be common to all individuals.

### Implementation

The freely available software WinBUGS v1.4.1 [15] is used to implement the hierarchical state-space model. The software uses Markov Chain Monte Carlo (MCMC) methods to estimate the joint posterior distribution of the model parameters. A total of 40 000 MCMC samples were generated in each of 2 chains, the first 20 000 were discarded as a “burn-in” and every 5th sample thereafter was retained to reduce autocorrelation, yielding 8 000 samples from the joint posterior. By deriving 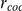 within the WinBUGS model, we obtain a marginal posterior distribution for each 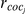 as well as their mean (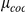). The model code is included in Appendix A.

### Data

**Figure 2:**
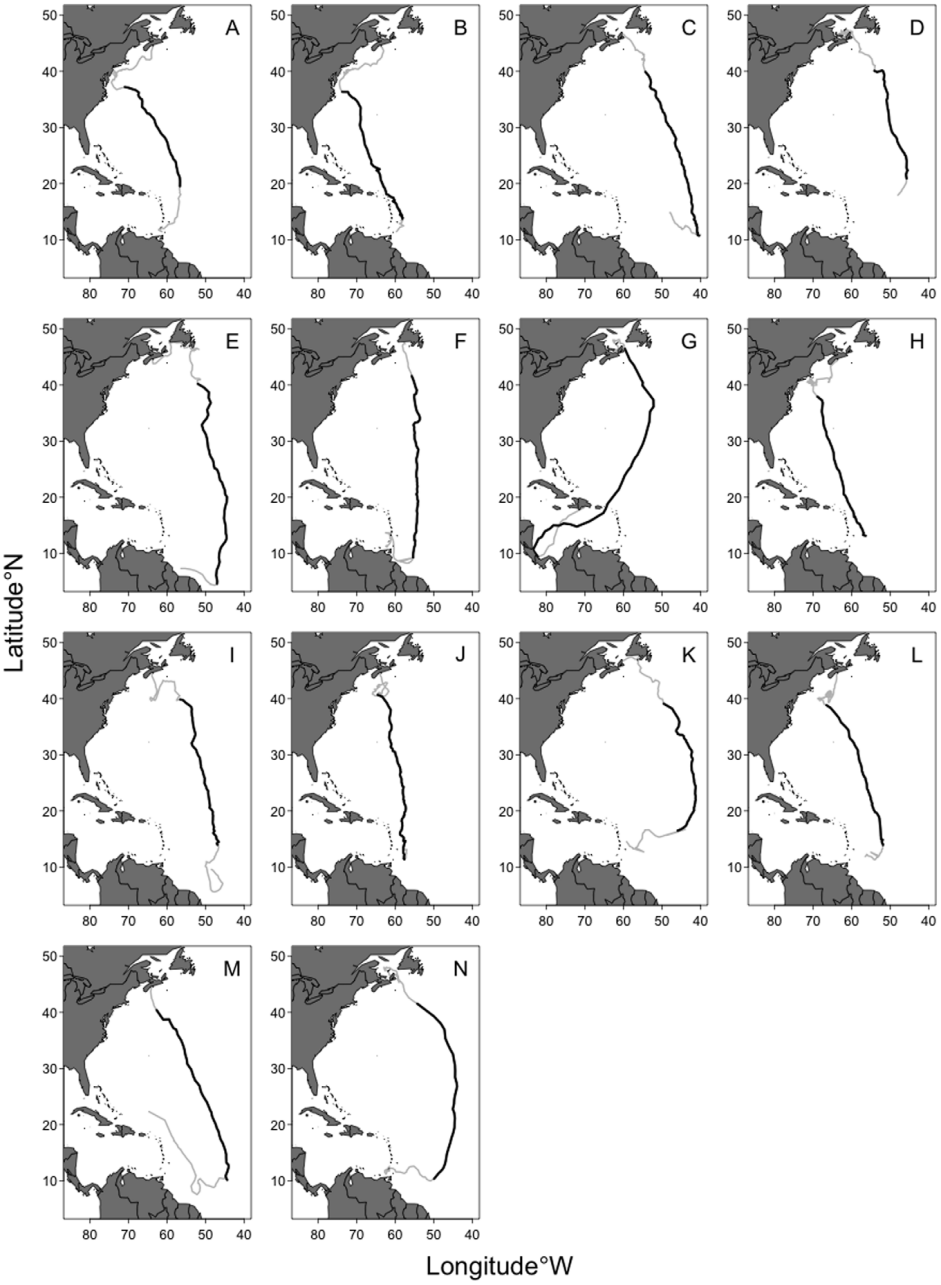
Maps of the fourteen leatherback turtle tracks using the raw Argos satellite data. Pathway segments used in the analyses are in bold. Panel labels correspond to the y-axis tick labels in Figure 5.

The data consist of southward migratory pathways of 14 adult leatherback turtles (7 male and 7 female; Figure 2) captured, equipped with Argos satellite transmitters, and released off the coast of Nova Scotia, Canada [2]. In order to fit our proposed model to data obtained with the Argos system, we require equally spaced observations in time. To obtain this data, hereafter referred to as *regularized*, we define a stepsize of 24 hours which is reasonable given that we are modelling migratory pathways than span approximately 3 months. This stepsize results in a series of 1-day windows within each of which we must then obtain a two-dimensional estimate of location. We utilize the Minimum Covariance Determinant (MCD) estimator [16] which yields a robust location estimate for each window that is not highly influenced by outlying locations caused by the limitations of Argos. In cases where we have less than four locations within a window, and hence the MCD is not computable, we take the coordinate-wise median (also robust) as our estimate of location. Note that one could also include the data quality measures provided by Argos along with each location estimate [17]. However, at present we believe that the reported standard errors for each location class are in need of refinement.

**Figure 3:**
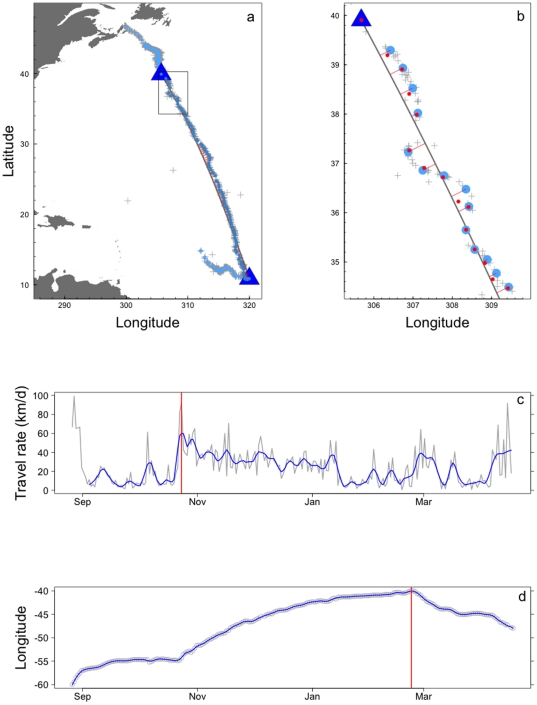
Example of how the Great Circle (GC) route and regularized deviations are obtained for turtle C. (A) Panel illustrating the full set of observed locations (grey pluses), the MCD-regularized locations (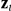, blue circles), the calculated GC route (grey line) and the deviations (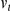) between the GC route and the MCD-regularized locations (red lines). The blue triangles indicate the determined start and end points of the GC route. (B) Enlargement of the region contained by the box in panel (A). The estimated true locations (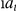, red circles) were derived from the estimates of 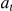. (C) Robust nonlinear smoother (blue) fit to MCD-derived travel rates (grey). The vertical red line denotes the peak travel rate associated with the onset of migration. Note that the smoother was not fit to the first week of data because these elevated speeds are likely a post-tagging behaviour. (D) Robust nonlinear smoother (blue) fit to MCD-derived longitudes (grey). The vertical red line denotes a large change in direction of travel potentially associated with the cessation of migration.

Having regularized our data and then calculated the observed deviations that we fit using our SSM (Equations (1) and (2)) we can now be more concise about the measurement error and process error distributions for this particular application. Given that most of the measurement error has been removed by the MCD (see Figure 3, subpanel (b)) it is reasonable to assume that the measurement errors are independently and identically *normally* distributed with mean 0 and variance 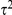. The process errors 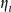, on the other hand, may still be inflated on occasion by the necessity for an animal to deviate from course, not due to limitations in navigation, but rather due to exogenous (e.g., deflection by ocean currents) or endogenous (e.g., the need to forage) factors. It is important that our model be robust to these larger errors so that our resulting estimates are not inflated by this sort of behavior. We therefore assume that the process errors follow a *generalized* 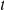 distribution with mean 0, variance 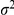 and 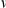 degrees of freedom. Such a distribution (when 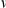 is small) allows for longer tails (a usual type of deviation) thereby making outliers less *unlikely* under the model.

There is considerable variability in leatherback turtle migratory behaviour as seen in Figure 2, in part this is determined by age, sex and, for adult females, whether they reproduced the previous year [2]. All leatherbacks migrate southward from Canadian waters, presumably as water temperature and productivity decline in the late fall and early winter [18], however, not all leatherbacks migrate to nesting beaches in Caribbean and Gulf of Mexico [2]. Adult females that reproduced in the previous year and juveniles tend to migrate southward to the Caribbean or northeast of Brazil and then migrate back to Canadian waters without reaching any nesting beaches. In order to have a sufficiently general method that deals with this variability in migration patterns, we chose to define the migration start and end points based on straightforward properties of the movement paths: travel rate and direction. We note that individuals that migrated to nesting beaches generally slowed down and changed direction several 100 km from their destinations. These turtles may be switching navigational modalities as they near their nesting beaches [19] and, as a consequence, we chose to remove these path segments from our analyses as their inclusion would likely bias the circle of confusion estimate.

Panels (c) and (d) of Figure 3 illustrate how the migratory portion of Track C was determined (the same procedure was used for all tracks). Since James *et. al.* [2] suggest that there is a peak in rate of travel associated with departure from northern foraging areas, we fit a robust nonlinear smoother to the times series of successive daily distances traveled (as calculated from the regularized data). We use this to identify the peak in travel, that is, the assumed start point of migration. With the start point of each migratory portion now available for each turtle, it remains to determine the end point of each migration. For the fourteen leatherbacks of interest here, their behavior is characterized by a migration southward followed by more localized behavior associated with foraging and/or breeding site selection. These latter behaviors are characterized by frequent changes in direction and provide a means with which to determine when migration ceases for an extended period of time. Again we use a robust nonlinear smoother, this time applied to changes in longitude. By examining these change points in conjunction with the corresponding tracks the migratory end points are identified. An alternative approach here would be to use a more complex SSM which included behavioural switching. Migratory and non-migratory behaviour could then be identified within the model with only migratory portions involved in the estimation of the circle of confusion.

### Analysis

Our hierarchical state-space framework allows us to specify informative prior distributions for those features of the model that we know something about. Much has already been documented about measurement errors for Argos location classes. Vincent *et al.* [17] report measurement error estimates over the 6 Argos location classes for both latitude and longitude. We obtained a rough idea of the magnitude of the measurement errors in our application by taking the mean of the measurement error estimates (68%-ile, non-filtered) as well as performing a sensitivity analysis to ensure our estimate for 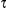 was appropriate.This information was incorporated into our analysis by specifying a normal prior distribution for 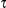 with a mean of 2 km and a standard deviation of 0.02 km.

In order to formally tests hypotheses about leatherback turtle migration we use the Minimum Posterior Predictive Loss Statistic, 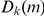 [10], to compare models 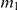 and 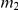 corresponding to the competing hypotheses. Specifically we use equation (6) from Gelfand and Ghosh [10] corresponding to squared error loss and compute 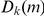 for each model with several values of 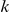, say 1, 3, 9, and 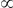. As they recommend, we then use these values to determine which model has better support. We present 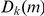 results using 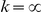, but model ranks were the same regardless of the value of 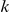 chosen. Note that the equation for 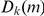 is made up of two parts. The first represents a squared error goodness of fit in which the predictive mean is compared to the observed value while the second term measures the predictive variances and can be viewed as a penalty term. We decide on the model with the smallest value of 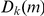 keeping in mind that if we argue for the more complicated model, the difference in 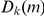 should be relatively large in the appropriate scale of the problem. In applying 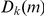, our basic observation is the 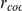 for each animal computed from their individual regularized track.

Using the methodology described above we test two distinct hypotheses regarding leatherback turtle navigation ability during the southward phase of their migratory cycle. First, we test whether male and female leatherbacks have different sized circles of confusion by fitting two hierarchical models to the deviations: (1) male and female deviations pooled (Common) and (2) male and female deviations separated (Separate). In the second model, we estimate 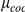 and associated parameters separately for males and females. A difference in 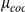 between males and females may reflect differences in navigational ability or the navigation cues relied upon.

**Figure 4:**
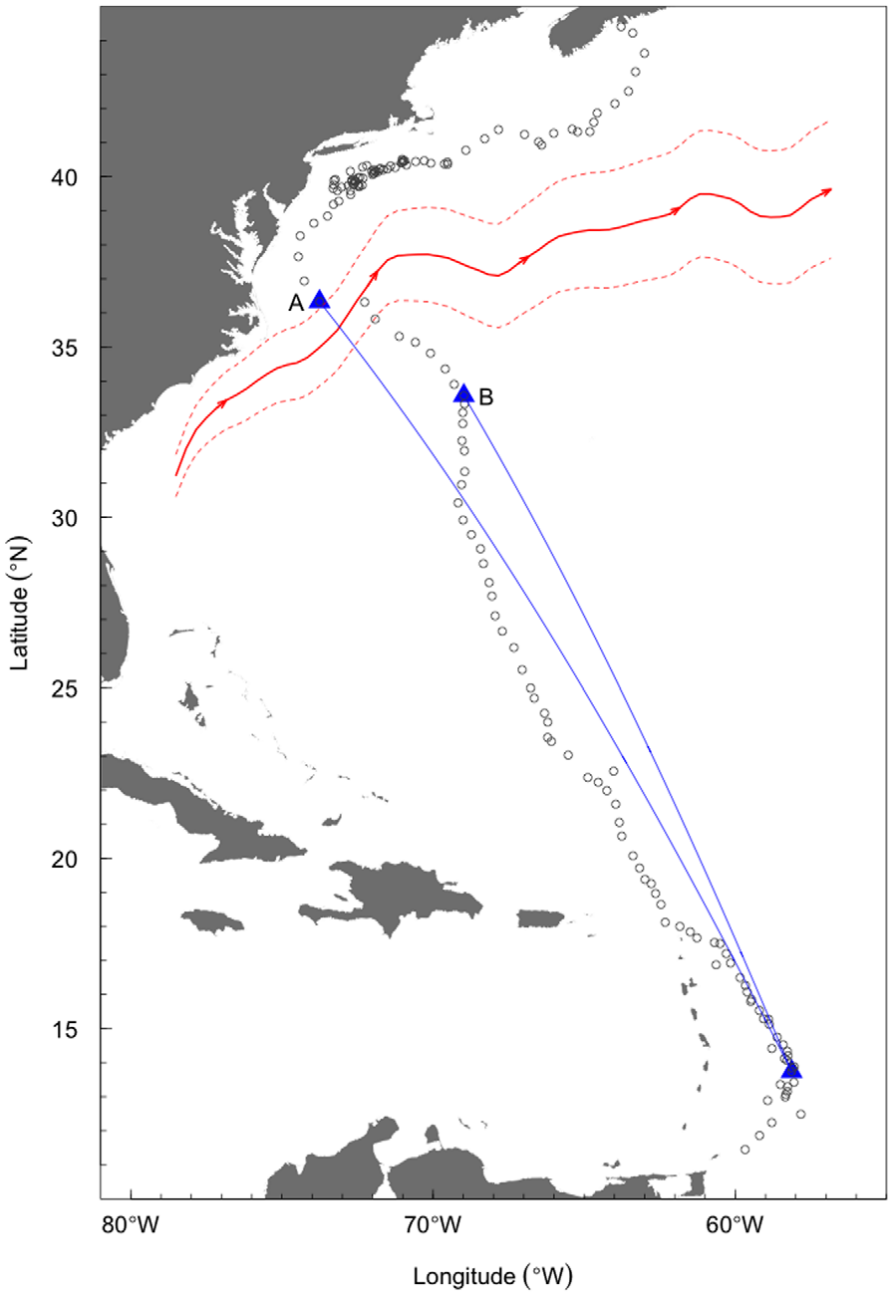
Plot of a MCD-regularized male leatherback turtle track indicating the Great Circle routes (blue) defined by starting positions before (A) and after (B) encountering the Gulf Stream. The mean position of the Gulf Stream (solid red line) +/− 2 sd (dashed red lines) aligns well with the rapid eastward movement of the turtle starting at point A.

Second, we test whether male leatherbacks compensate for drift when crossing the Gulf Stream current, a strong current that originates in the Gulf of Mexico and flows northeast, parallel to the eastern coast of North America, by following a new great circle route after crossing. Several turtles were observed to drift eastward while crossing the Gulf Stream, this is most noticeable in Figure 2 (turtles A, B, E, and G). For clarity, Figure 4 illustrates this observation for Turtle B. It is possible that turtles “re-calculate” their migratory route after being deflected from their original course. If this is the case, circle of confusion estimates should be smaller when fitting to deviations calculated after the turtles crossed the Gulf Stream than for deviations calculated before the turtles crossed. To test this hypothesis, we fit the same model to the deviations from the full male tracks (Figure 2) and to the deviations calculated only after the turtles had crossed the Gulf Stream. We compare 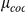
's from these two fits and again use the 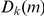 statistic to determine which hypothesis has greater support.

We tested whether the circle of confusion model could be better described as a random walk tied down at both ends. As such, deviations at each of these ends might be smaller than expected which would lead to an under-estimate of the circle of confusion. Such a situation could arise if the turtles are engaging in different movement or navigation processes near the start and end of the pathway segments that we analyzed. To investigate, we removed the first and last 2.5% of the deviations from each pathway and re-ran our analysis and compared 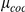 estimates to those from the full pathway segments.

## Results

### Sex differences

**Figure 5:**
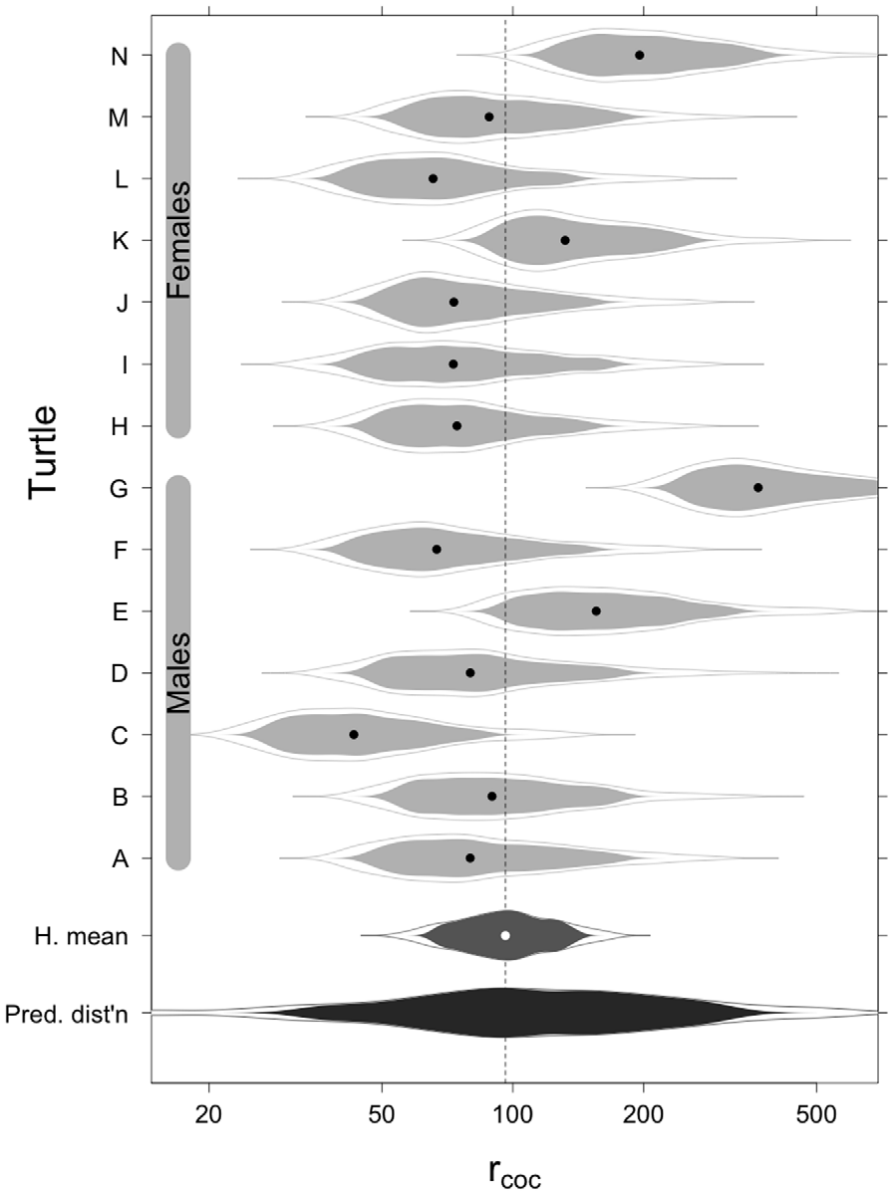
Raindrop plots [**31**] indicating posterior medians with 95% credible limits for the circle of confusion 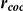 obtained from the hierarchical model fit to 7 male and 7 female leatherback turtle tracks. The larger raindrops in the bottom two rows are hierarchical summaries. The medium gray raindrop denotes the hierarchical means and the black raindrops denotes the Bayes predictive distribution.

Comparison of 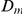 for the Common and Separate models suggests that male and female leatherbacks have similar navigation abilities (Table 1, Figure 5). On average, turtles had an 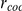 of 96.2 km (Figure 5), males had a 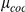 of 98.7 km and females had a 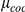 of 75.3 km (Table 1). The high 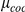 estimate for males is largely driven by a single turtle (G, Figure 5) that migrated from Nova Scotia to waters off a nesting beach in Panama, whereas all other turtles migrated to the eastern Caribbean (Figure 2). Estimates of 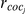 for individual turtles indicate a range in navigation ability of 43 to 367 km. The 367 km estimate was turtle G, and excluding this extreme estimate, we found that 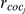 ranged between 43 and 196 km.

**Table 1:** Posterior quantiles of the key parameters for the Common and Separate models.

Model	Parameter	Posterior Quantiles		
		2.5%	50%	97.5%
Common	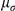	0.0014	0.0021	0.0031
	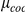	53.4300	96.1550	173.4125
	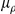	0.9786	0.9900	0.9962
	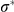	0.0003	0.0022	0.0056
	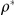	0.9592	0.9923	0.9995
	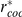	10.6680	108.3000	652.2275
	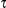	0.0003	0.0003	0.0003
	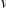	2.6580	3.4055	4.4001
Separate	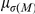	0.0008	0.0025	0.0045
	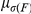	0.0011	0.0019	0.0029
	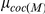	29.7582	98.7250	222.2725
	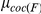	36.9185	75.2650	154.2675
	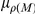	0.9670	0.9868	0.9955
	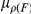	0.9676	0.9874	0.9956
	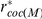	9.3615	133.6500	768.5625
	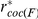	13.5970	86.0100	371.6075
	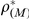	0.9455	0.9896	0.9995
	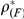	0.9482	0.9902	0.9991
	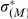	0.0002	0.0030	0.0093
	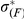	0.0003	0.0019	0.0045
	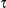	0.0003	0.0003	0.0003
	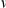	2.6829	3.4805	4.5007

Both models were fit to 14 leatherback turtle tracks, 7 males and 7 females. Parameters are estimated separately for the sexes in the Separate model. Asterisked parameters, e.g., 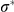, denote the posterior predictive distributions. The Minimum Posterior Predictive Loss statistic [10], 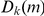, values were 13,825 and 14,564 for the Common and Separate models, respectively.

Hierarchical and individual-level estimates of 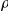 reveal a high degree of autocorrelation in the deviations from great circle routes (Table 1, 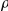 ranged between 0.96 and 0.99) and are consistent with our observation that the turtles tend to remain on one side or the other of the most direct route.

### Drift compensation

Comparison of 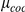
's from the model fit to the full deviations and the model fit to deviations after crossing the Gulf Stream suggest that male leatherbacks do compensate for eastward drift by following a new great circle route. The 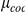 were 140.5 km and 106.8 km for the full track and the track after the Gulf Stream, respectively (Table 2). In addition, there was greater support for the model fit to deviations calculated after the Gulf Stream (see 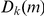 in Table 2).

**Table 2:** Posterior quantiles of the key parameters for the hierarchical model fit to 7 male leatherback turtles.

Dataset	Parameter	Posterior Quantiles		
		2.5%	50%	97.5%
Full track	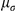	0.0013	0.0037	0.0064
	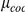	47.5227	140.5000	364.0000
	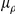	0.9668	0.9852	0.9974
	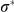	0.0003	0.0042	0.0117
	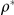	0.9617	0.9858	0.9984
	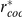	11.7397	163.2000	680.2200
	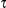	0.0003	0.0003	0.0003
	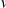	2.1050	2.7255	3.7350
After GS	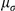	0.0011	0.0031	0.0055
	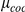	36.9385	106.8000	317.4025
	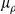	0.9546	0.9825	0.9976
	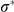	0.0003	0.0035	0.0102
	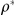	0.9484	0.9832	0.9984
	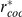	9.7166	124.8000	557.9050
	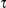	0.0003	0.0003	0.0003
	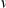	2.0240	2.4140	3.3901

Deviations were calculated for each of the full tracks (Full track) and for only those segments of the tracks after the turtles had crossed to the southern side of the Gulf Stream (After GS). Asterisked parameters, e.g., 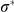, denote the posterior predictive distributions. The Minimum Posterior Predictive Loss statistic [10], 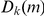, values were 16,345 and 8,789 for the Full track and After GS model fits, respectively.

The degrees of freedom (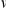) of the process error distribution is estimated to be approximately 2.9 or less for all models. This small value of 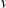 supports our decision to use a (generalized) 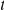 distribution rather than a normal distribution for the process error. A 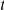-distribution with small 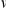, has much heavier tails than that of a normal distribution, and, as such, allows outlying process errors to have far less impact on parameter estimates (in this case the 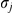
's) than would otherwise occur. This approach is helpful in down-weighting the influence of large transient deviations, for example changes imposed by switches between opportunistic foraging and migration, from the great circle route on 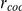 estimates while allowing for moderate deviations. The approach does not appear to minimize the effect of longer-term, moderate deviations such as those generated, for example, from persistent physical forcing like the eastward drift in migration as a result of crossing the Gulf Stream.

Finally, we find no evidence to suggest that the results are driven by data points near the start and end points of the migration track, 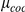 only changes slightly when the first and last 2.5% of the data are removed. In addition, individual level parameter estimates are nearly identical to those in Figure 5.

## Discussion

We present a hierarchical framework for thinking about a question that has not been quantitatively addressed: how well do animals navigate? Our approach makes the assumption that animals attempt to migrate along the most direct route to their destination; in this case the great circle route between foraging sites in the Northwest Atlantic and breeding sites in the Caribbean. By taking a hierarchical Bayes approach to modelling navigation ability we implicitly assume that the individuals exhibit some degree of similarity in their navigation ability. Specifically, the 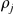
's come from a common distribution and similarly for the 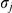
's. The hierarchical model is the formal representation of these assumptions, with the goal being to estimate not only the model parameters at the individual level (the 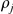
's and 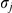
's) but also those at a broader organizational level (the hyper-parameters; 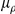, 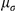, and 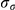). There are two principle advantages of this hierarchical approach over modelling the pathways separately. First, hierarchical models combine information from all of the tracking data to estimate parameters at both the population and the individual levels [20]. Combining information in this way leads to more efficient parameter estimation. Second, hierarchical Bayes models allow quantification of all the uncertainty in the parameter estimates via the predictive distribution, also refered to as the *induced prior* [21]. The predictive distribution, as its name suggests, forms the basis for prediction of navigation ability for unobserved individuals. For example, we can also use the predictive distribution as our current best estimate of the population-level variability in leatherback navigation ability.

Our estimate, that leatherback turtles, on average, know their position to within 96 km suggests that their navigational sense during the pelagic phases of their migration does not have to be highly accurate if they use other cues as they near their destination. This conclusion is consistent with analytical and simulation work of Kendall [8] on Manx shearwater migrations across the Atlantic. A key result from Kendall's work was that for migration to be efficient, an animal's circle of confusion need only be slightly smaller than the range at which other cues can be used for orienting toward a destination. For example, it has been suggested that green turtles (*Chelonia mydas*) use one set of cues to navigate from the Brazilian coast to the vicinity of Ascension Island and thereafter rely on olfactory cues [22]. It is possible that leatherbacks use a more complex strategy because there is no single, isolated island to which migrations from northern waters are directed; however, individuals do appear to exhibit fidelity to breeding/nesting areas [23].

Clearly, refinements can be made to our approach. For example, if there are persistent forces, e.g., oceanic currents, that cause migrating animals to undergo long-term deviations from their intended course, our circle of confusion estimate will overestimate the true value. It is easy to see the influence of the Gulf Stream on the two leatherbacks that approached Cape Hatteras (Figure 2 A, B). Although the method is robust to short-term deviations by assuming the process error follows a 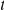-distribution, the circle of confusion estimates could be further improved by making use of oceanographic data to include longer-term effects in the model. Accounting for environmental effects implies that more details of the movement process would have to be accounted for by the model [24], hence sacrificing some of the generality of the current approach.

The underlying assumption of great circle navigation could be replaced with other models. Migrating birds often make migrations in stages, approximating the most direct route on each stage but yielding a less direct path from origin to the final destination [25]. In this case, the great circle navigation ability could be assessed along each stage of an individual's migratory track by adding a within-individual level to the hierarchical model. This example illustrates how the approach can be modified to suit more complex situations when additional information on migratory routes and migratory behaviour is available. The simple approach illustrated here makes minimal assumptions, is generally applicable and would be preferred when little information other than the tracking data are available. As the use of GPS data becomes more ubiquitous, other approaches such as dynamic GLMs and the Kalman filter could be used to fit the Circle of Confusion model to these more precise data. However, in many terrestrial settings, GPS data are also prone to occasionally large errors due to interference from vegetation and topography [26], [27]. In these situations a Bayesian (via MCMC) approach may still be most appropriate for estimating navigation ability as the occasionally large errors can be handled easily by assuming t-distributed measurement errors. Additionally, the Bayesian approach allows great flexibility in specifying hierarchical models such as one fit here.

Many analyses of navigation ability have been conducted at the individual level [28] and usually based on summary statistics derived from tracking data [25], from releases of manipulated animals [29] or from laboratory experiments [30]. However, much more information is available from the analysis of full migratory pathways, the challenge has been in dealing with the sequential nature of tracking data and in combining information across multiple individuals. The hierarchical state-space approach presented here addresses these challenges, providing a tool for ecologists to test hypotheses about migratory behaviour and navigation ability from ubiquitous electronic tracking datasets.
